# Developing IBD counsellors in low- and middle-income countries: bridging gaps in patient care

**DOI:** 10.1016/j.eclinm.2025.103218

**Published:** 2025-04-24

**Authors:** Arshdeep Singh, Arshia Bhardwaj, Riya Sharma, Vandana Midha, Ajit Sood

**Affiliations:** a Department of Gastroenterology, Dayanand Medical College, Ludhiana, Punjab 141001, India; b Department of Internal Medicine, Dayanand Medical College, Ludhiana, Punjab 141001, India

**Keywords:** Inflammatory bowel diseases, IBD counsellors, Health equity, Healthcare delivery, Global health

## Abstract

The global burden of inflammatory bowel disease (IBD) is progressively increasing, with a particularly sharp rise in newly industrialized and resource-limited settings. These regions face unique and pressing challenges in IBD care, including a shortage of trained specialists, delayed or missed diagnoses, financial and geographic barriers to access, and the persistent stigma surrounding the disease. Furthermore, cultural dynamics; especially the prominent role of family in healthcare decisions; profoundly influence patient engagement, treatment adherence, and overall outcomes. However, current healthcare models and global guidelines are largely shaped by Western systems that prioritize individual patient autonomy and may not fully align with the sociocultural realities of resource-limited settings. This viewpoint aims to highlight the need for culturally contextualized, scalable solutions to improve IBD care. Specifically, we propose the development and integration of IBD counsellors as a novel and pragmatic approach to bridge existing gaps in care. These counsellors, trained in the nuances of IBD and sensitive to local sociocultural norms, can serve as critical intermediaries; facilitating communication among patients, families, and providers; supporting adherence and follow-up; and offering tailored psychosocial and dietary guidance. By presenting this model, we seek to stimulate discourse around innovative, culturally adaptive strategies and advocate for policy-level recognition and investment to promote health equity in IBD care globally.

**Search strategy and selection criteria.**

References for this Viewpoint were identified through a comprehensive search of PubMed using the following search terms: “Inflammatory Bowel Diseases,” “Resource-Limited Settings,” “Counsellors,” “IBD Nurse”, “Follow-Up Studies,” “Clinical Decision-Making,” “Family Relations,” “Diagnostic Errors,” “Health Personnel,” “Health Services Accessibility,” and “Communication.” The search covered literature from inception until December 2024. Additionally, relevant articles were identified through a review of the authors’ files. Only English-language publications were considered. The final reference list was curated based on originality and relevance to the overarching scope of this Viewpoint.

## Developing IBD counsellors in low- and middle-income countries: bridging gaps in patient care

Inflammatory bowel diseases (IBD), encompassing Crohn's disease and ulcerative colitis, are chronic, debilitating inflammatory disorders of the gastrointestinal tract marked by a relapsing remitting course. Globally, the prevalence of IBD has risen markedly, with reported cases increasing from 3.32 million in 1990 to 4.90 million in 2019.^1^^,^^2^ While the incidence of IBD in Western countries has stabilized, developing regions across Asia, South America, and the Middle East have witnessed a substantial surge in disease burden.^1^^,^^3^, ^4^, ^5^, ^6^

At the turn of the 21st century, the incidence and prevalence of ulcerative colitis in India were estimated to be 6.02 and 44.3 per 100,000 inhabitants, respectively.^7^ Currently, there are no population-based epidemiological studies assessing the disease burden of Crohn’s disease in India. However, hospital-based cohort studies indicate a rising trend in its occurrence over the years.^3^^,^^4^ Comparative analyses of incidence and prevalence rates suggest that, among Asian countries and low- and middle-income countries (LMICs), India bears the highest disease burden.^8^ IBD is further associated with significant morbidity, including complications such as malnutrition, strictures, fistulae, and malignancies. The disease profoundly impacts health-related quality of life (HRQoL), contributing to mental health challenges, social isolation, and financial hardships due to the high costs of treatment and frequent hospitalizations.^9^, ^10^, ^11^, ^12^

Despite the rising burden of IBD, healthcare systems in LMICs remain underprepared to manage the unique challenges posed by IBD.^13^^,^^14^ There is a significant shortage of specialists trained in gastroenterology and IBD care, leading to an overwhelming patient load on the available doctors.^15^ While there is no official communication on the exact number of practicing gastroenterologists in India, the Indian Society of Gastroenterology has approximately 4200 members. Given India's estimated population of 1450 million in 2025, this corresponds to 0.000003 gastroenterologists per 100,000 inhabitants, a figure significantly lower than that observed in developed countries. For reference, the number of gastroenterologists per 100,000 inhabitants in 2007 was 3.9 in the United States, 3.48 in France, 2.1 in Australia, 1.83 in Canada, and 1.41 in the United Kingdom, with subsequent improvements expected in these ratios over time.^16^ Interestingly, and ironically, the number of practicing family physicians in India is rapidly declining, despite the country’s pressing public health needs. The previous generation of general practitioners is aging, and no new family physicians are establishing practices in urban or rural areas.^17^ Moreover, a pervasive lack of understanding about IBD exists among healthcare professionals, patients, and the general public.^18^, ^19^, ^20^, ^21^ Most patients are uncertain of their condition, its chronic nature, and the importance of adherence to treatment and follow-up care. Misdiagnosis or delayed diagnosis is common, with symptoms often attributed to more familiar conditions like infectious diarrhoea or irritable bowel syndrome.^13^ This gap in knowledge not only hinders effective disease management but also contributes to stigma and misconceptions. Overburdened healthcare infrastructure further complicates the situation, leading to rushed consultations and inadequate time for patient education. Many patients, unable to access specialized IBD care, turn to unqualified practitioners or receive inappropriate treatments, exacerbating their condition.^22^^,^^23^ Geographic and financial barriers also restrict access to IBD physicians and advanced therapies, especially in rural or remote areas. A health economic study on IBD from India reported that the annual median cost per patient with ulcerative colitis and Crohn’s disease in remission was Indian Rupee (INR) 43,140 (IQR: INR 34,357–51,031) and INR 43,763.5 (IQR: INR 32,202–57,372), respectively. In contrast, the corresponding costs for patients with active disease were INR 52,436.5 (IQR: INR 49,229–67,567.75) and INR 72,145 (IQR: INR 49,447–92,212), respectively.^24^ A significant proportion of these expenses are out-of-pocket due to the absence of universal health insurance coverage. In 2024, India’s estimated annual per capita income was approximately INR 1,84,000, indicating that the cost of IBD management accounts for nearly 25–40% of the annual per capita income. Furthermore, unlike other non-communicable diseases such as cardiovascular diseases, cancers, diabetes, and hypertension, IBD lacks dedicated healthcare policies, translating into a lack of structured screening, treatment protocols, and support mechanisms for patients.

In Western countries, the roles of IBD nurses, physician assistants, and dietitians are established, but limited to predefined scopes of practice. However, there is a need to address the broader, multidisciplinary needs of IBD patients, especially in regions where these dedicated IBD nurses or equivalent roles are not available. Even within western healthcare systems, these services are not universally accessible. Also, there remains a pressing need for enhanced training and collaboration with specialized mental health professionals who understand the unique psychological challenges faced by individuals with IBD.

These unmet needs highlight the necessity of a dedicated intermediary who can simplify complex medical concepts, provide continuous education, support patients and their families in navigating their IBD journey, and facilitate effective communication between patients and healthcare providers. Therefore, integrating a specialized healthcare professional, an IBD counsellor, into healthcare systems is essential.

## What are the potential roles of an IBD counsellor?

IBD counsellors will serve as a crucial link between patients and healthcare providers. A primary role of the IBD counsellor will be to maintain regular and frequent contact with patients, ensuring continuous support throughout their disease journey. By fostering a strong, ongoing relationship, the counsellor will act as a trusted guide and advocate, much like having adopted the patient.

The following section outlines the key responsibilities of IBD counsellors and their impact on patient care.

### Bridging the gap between doctors and patients

IBD counsellors will play a pivotal role in improving communication between patients and healthcare providers. By acting as intermediaries, they will help address patient concerns, clarify medical jargon, and ensure patients fully understand their condition, including the disease, its natural course, treatment options, current health status, and how to cope up with the diagnosis of IBD. This will foster trust, encourage shared decision-making, and promote patients as partners in disease management.

### Monitoring and early detection of symptoms

The IBD counsellors will maintain ongoing communication to track symptoms, identify signs of relapse, and guide patients on when to seek medical help. In LMICs specialized IBD care is often limited and concentrated in urban hospitals, with restricted access to healthcare services due to overburdened systems and geographic barriers. As a result, many patients experience delays in receiving timely medical attention. The integration of IBD counsellors into multidisciplinary teams presents a practical and effective solution to enhance patient care and improve disease management. Regular monitoring of symptoms, such as diarrhea, blood in stools, abdominal pain, urgency, fatigue, weight loss, and fever, will enable early identification of relapse or complications. By recognizing an impending relapse, IBD counsellors can facilitate timely interventions such as medication adjustments, imaging, or endoscopy to confirm disease activity and guide treatment. This proactive approach will not only prevent complications but also reduce hospitalizations, ensuring effective and personalized care for patients.

### Medication compliance

Non-adherence to treatment is a major challenge in IBD management. IBD counsellors will educate patients about the importance of adhering to prescribed regimens, emphasizing how complying with treatment helps maintain remission and reduces relapses. An important role would be to also help the patient understand rectal or topical therapies. Another key focus will be on mitigating the tendency of patients to decrease or skip medications once symptoms improve, a behavior that significantly increases the risk of relapse.^25^ By fostering a better understanding of the long-term benefits of adherence and supporting patients through personalized guidance, IBD counsellors are expected play a vital role in achieving the goals of medication adherence and sustained disease control.^26^

### Facilitating follow-up care

This approach aligns with the concept of the IBD home, a patient-centered care model designed to provide continuous, comprehensive, and accessible care for individuals with IBD.^27^^,^^28^ IBD counsellors will play a pivotal role in facilitating follow-up care, ensuring that consultations are timely, efficient, and cost-effective.

Key services under this model will include telemedicine and online follow-up sessions, which are particularly beneficial for patients in rural or remote areas with limited access to specialized care. While we acknowledge that telemedicine services may not be feasible in all regions due to limited availability of high-speed internet connectivity, mobile networks are often accessible, allowing for the use of mobile health technologies such as WhatsApp, SMS-based reminders, and voice calls to maintain communication between patients and healthcare providers through IBD counsellors.^29^, ^30^, ^31^

Additionally, IBD counsellors can function within a hub-and-spoke model to enhance follow-up care. Rather than facing long waiting periods for direct communication with physicians, patients can engage with IBD counsellors remotely via phone calls or mobile applications, while scheduling periodic in-person visits at nearby IBD centers. IBD counsellors can further relay patient concerns to specialists and provide timely guidance, thereby improving access to care despite geographical barriers. This structured approach not only reduces travel-related burdens and associated costs but also optimizes patient management, ensuring better adherence to treatment plans and improved disease outcomes.

### IBD education, family support, and counseling

In many traditional societies, family involvement is central to healthcare decisions. Educating both patients and their families about IBD enhances informed decision-making and fosters a supportive home environment. Educating both patients and their families about the complexities of the disease is essential for informed decision-making and improving quality of life.

### Transition from pediatric to adult IBD care

Transitioning from pediatric to adult IBD care can be challenging in settings where family dynamics play a strong role in healthcare decisions. IBD counsellors can guide young patients and their families through this shift, emphasizing self-management, independent decision-making, and effective communication with adult healthcare providers.^32^^,^^33^

### Psychosocial support

Mental health counselling is critically important in LMICs, where stigma, cultural barriers, and a severe shortage of trained professionals significantly limit access to care.^34^ IBD, per se, is also associated with increased rates of anxiety and depression, yet mental health support remains limited.^35^ Integrating mental health services into primary healthcare through task-shifting and training non-specialist providers is a pragmatic and effective approach. IBD counsellors will be trained to screen patients for symptoms of anxiety, depression, and stress, provide basic coping strategies, and facilitate referrals to mental health specialists when needed.

### Lifestyle and dietary guidance

Educating patients about the intricate relationship between diet and disease empowers them to make informed food choices, which can help reduce flares, improve symptom control, and enhance overall health and well-being.^36^, ^37^, ^38^ Collaboration between IBD counsellors and dietitians will help providing personalized nutrition plans tailored to each patient’s unique dietary triggers, nutritional requirements, and disease status. Physical activity has been shown to reduce inflammation, improve mental health, enhance immune function, and support overall physical fitness.^39^^,^^40^ With IBD counsellors encouraging patients to incorporate appropriate levels of exercise into their daily routine, improvements in quality of life can be expected. Smoking is a known risk factor for Crohn’s disease, worsening inflammation and increasing the likelihood of complications.^41^, ^42^, ^43^ IBD counsellors can educate patients on these risks and provide support for smoking cessation, helping to improve long-term disease outcomes. Similarly, poor sleep quality is associated with increased disease activity, fatigue, and a reduced ability to cope with IBD.^44^^,^^45^ Chronic inflammation and frequent nocturnal symptoms can disrupt sleep patterns, exacerbating stress and negatively impacting overall well-being. IBD counsellors can guide patients in adopting better sleep hygiene practices, managing stress, and recognizing when sleep disturbances may indicate worsening disease activity.

IBD counsellors will provide lifestyle recommendations based on the best available evidence while acknowledging the current limitations in guidelines. They will rely on expert consensus, emerging research, and patient-reported outcomes to guide recommendations. Additionally, IBD counsellors will be expected to continuously update their knowledge through professional training and collaborate with healthcare providers to ensure that the advice remains aligned with evolving evidence and individualized patient responses.

**Fig. 1:**
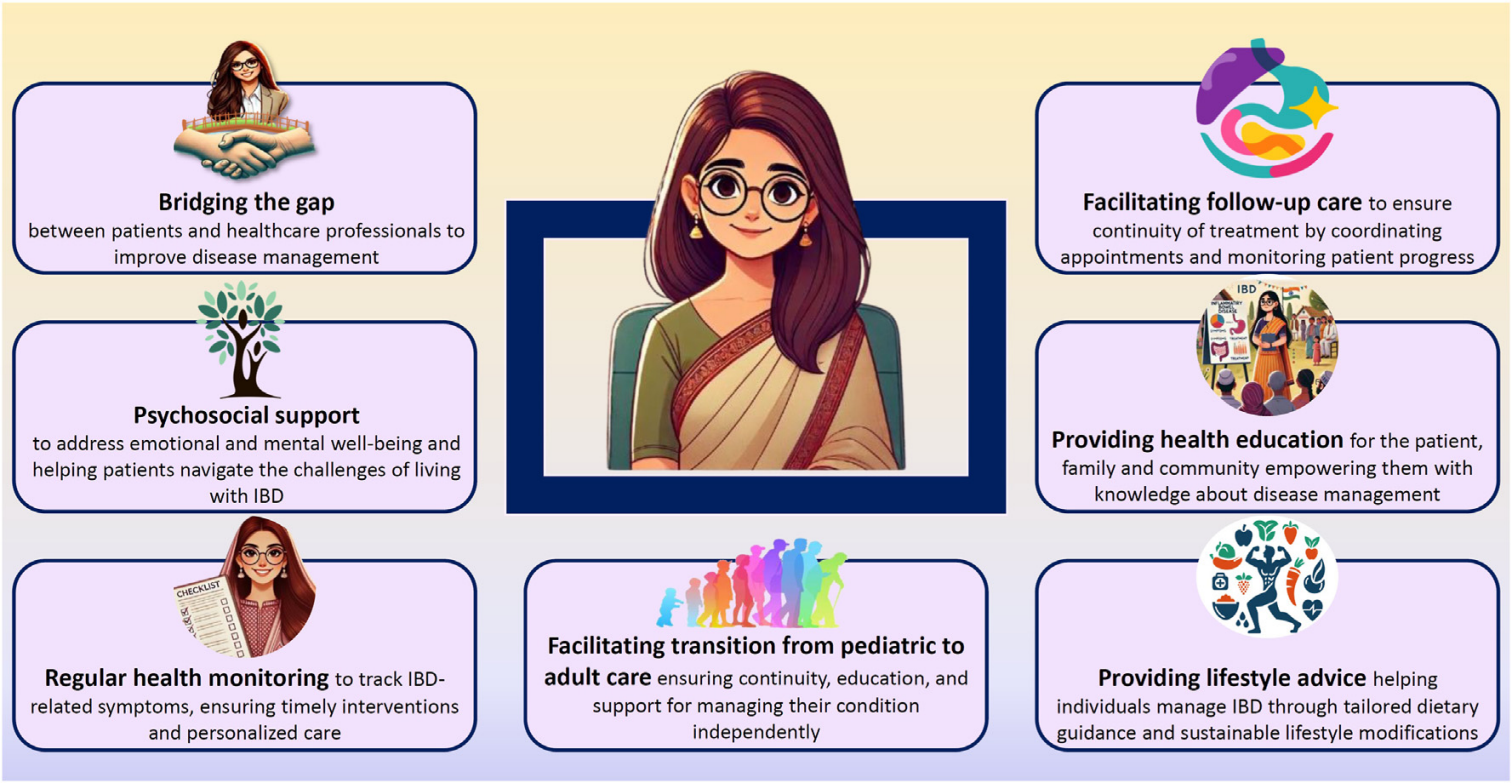
The multifaceted roles of Inflammatory Bowel Disease (IBD) counselors in addressing challenges and enhancing support.

To summarize, IBD counsellors, through their diverse responsibilities, will play a pivotal role in addressing the multifaceted challenges associated with IBD, and facilitate the adoption of a patient-centric care model (Fig. 1).

## How to develop IBD counsellors?

**Fig. 2:**
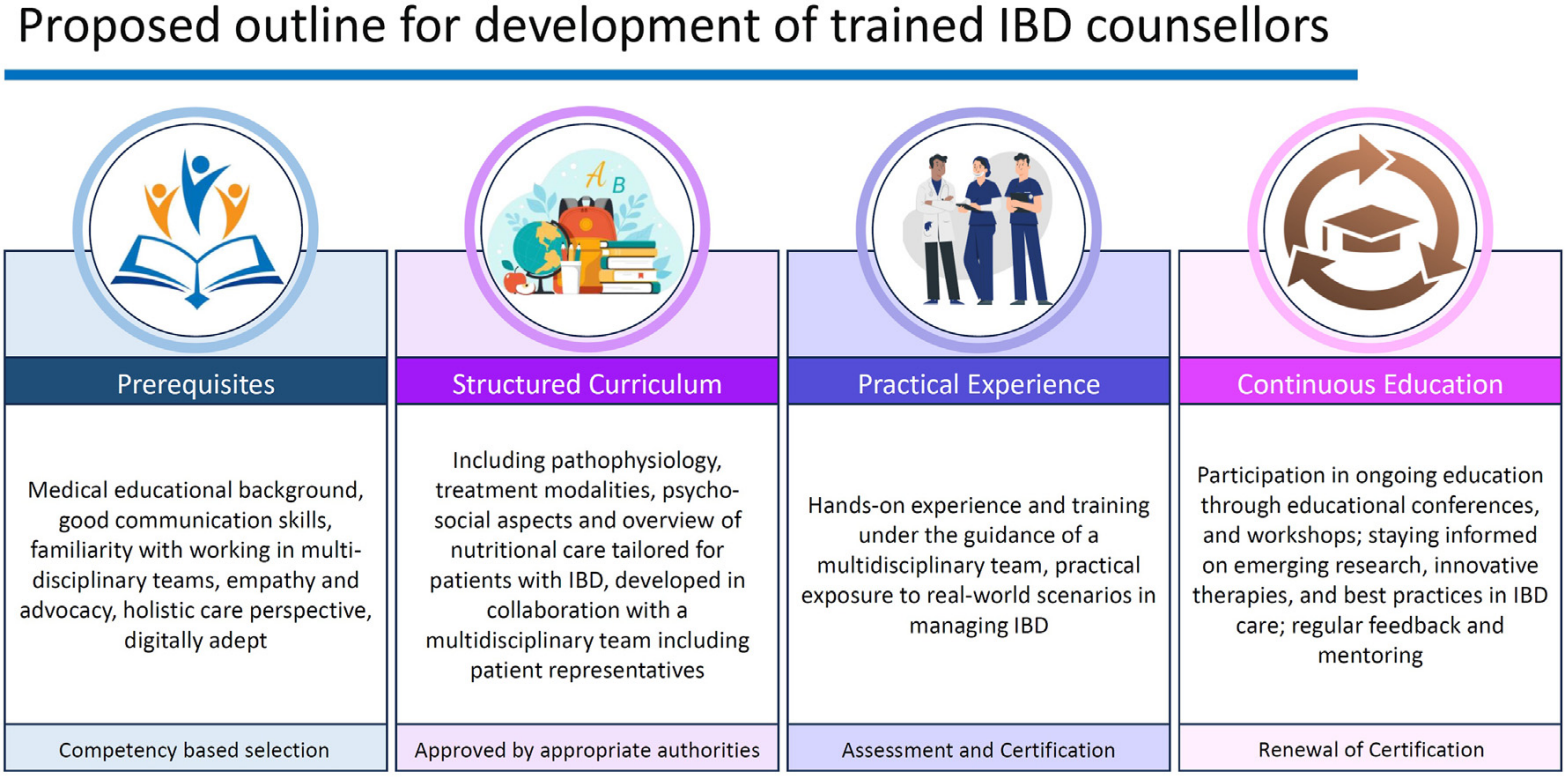
Proposed outline for development of trained Inflammatory Bowel Disease (IBD) counsellors.

The IBD Counsellor model builds upon and integrates the functions of IBD nurses, dietitians, mental health professionals, and family counsellors, into single, but accessible role, offering multidisciplinary, patient-centered approach to IBD care (Table 1). However, establishing this role necessitates targeted training and a well-structured framework to optimize patient outcomes (Fig. 2).

**Table 1:** Comparison of the IBD Counsellor with existing roles in IBD care.

	IBD nurse	Dietitian	Mental health professional	Family counsellor	Proposed IBD counsellor
Primary focus	Medical care Treatment coordination Patient education	Nutrition management Dietary counselling	Psychological support Mental health care	Family education Emotional support Coping strategies for caregivers	*Comprehensive patient care, integrating medical education, dietary advice, psychological support, and family guidance*
Scope of practice	Symptom management Medication administration Monitoring treatment response	Personalized diet plans Addressing nutritional deficiencies	Counselling Cognitive behavioural therapy	Helping families understand IBD	*Bridges medical, nutritional, psychological, and family support*
Patient interaction	Disease management and education	Nutritional guidance Meal planning	Addressing anxiety, depression, and stress related to IBD	Supporting families in coping with the emotional and social impact of IBD	*Central point of contact for patients and families, ensuring coordinated care*
Collaboration with other specialists	Works closely with gastroenterologists and other clinicians	Collaborates with physicians and patients for dietary interventions	Works independently or in conjunction with medical teams	Coordinates with IBD care teams, social workers, and psychologists to enhance family support	*Acts as a bridge between medical teams, dietitians, psychologists, and families*
Availability in healthcare systems	Well-established in Western healthcare systems Largely unavailable in developing and underdeveloped countries.	Common in Western healthcare settings and specialized IBD centers Limited availability in community healthcare settings.	Limited availability of IBD-specific expertise	Rarely available or highly limited in accessibility	*Designed to bridge gaps in care*
Potential impact	Enhances medical management and patient education	Improves nutritional health and dietary adherence	Addresses emotional and psychological burden	Strengthens family dynamics, reduces caregiver burden, and enhances patient support systems	*Provides holistic, well-rounded care by integrating all aspects of IBD management into a single, accessible role*

IBD, Inflammatory Bowel Disease.

### Prerequisites/core competencies

#### Educational foundation and medical knowledge

IBD counsellors should possess a medical background or come from allied specialties such as community health workers, nursing, dietetics, psychology, patient navigators, peer educators, or social work. This foundation ensures they have a solid understanding of the complexities of IBD, including its pathophysiology, disease course, complications, and treatment strategies. Given the shortage of healthcare personnel, the LMICs can implement a tiered approach to staffing IBD counselors by leveraging existing healthcare workers. The LMICs also have a large pool of unemployed allied healthcare professionals, presenting an opportunity to create jobs while strengthening the healthcare system. A phased implementation with structured training can ensure scalability without overburdening the existing workforce.

#### Communication skills

Effective counsellors must be active listeners, providing clear, empathetic explanations about the diagnosis, treatment options, and lifestyle changes. They should be adept at managing emotional distress, non-compliance, and culturally diverse patient needs. Additionally, they should be equipped to navigate discussions about complex treatment decisions, including the risks and benefits of long-term immunosuppression, escalation of therapy, and surgery. Addressing misconceptions about diet, stress, and complementary therapies in IBD is also crucial to prevent misinformation.

#### Digital competence

IBD counsellors should be proficient in telemedicine for online consultations and follow-up care. Familiarity with electronic health records is essential for documenting patient progress and ensuring effective communication with the healthcare team. Additionally, they should be trained in the use of digital symptom trackers, wearable health monitoring devices, and social media platforms (e.g., WhatsApp, Telegram, and patient-support groups, etc.) to facilitate patient engagement and real-time guidance on symptom management.

### Specialized certification

#### Structured curriculum

A structured curriculum should provide IBD counsellors with in-depth knowledge of the disease’s pathophysiology, diagnostic tools, treatment modalities, and the psychosocial and nutritional aspects of IBD care. Training should emphasize upon the following.

##### Pathophysiology of disease

Understanding complex interplay of genetic susceptibility, dysregulated immune responses, intestinal barrier dysfunction, and gut microbiota alterations.

##### Pharmacological knowledge

Understanding mechanisms of action, adverse effects, and monitoring requirements for IBD medications, including corticosteroids, 5-aminosalicylates, immunosuppressants, biologics, and small molecules.

##### Knowledge about diagnostic tools

Understanding the diagnostic tools allows to help patients interpret test results, alleviate anxiety about procedures, and reinforce the importance of timely investigations for optimal disease management. These include:

*Endoscopic assessments* to evaluate mucosal inflammation, assess treatment response, and detect complications like strictures or dysplasia.

*Imaging modalities* for assessing small bowel involvement, complications such as fistulae or abscesses, and disease activity in patients where endoscopy is not feasible.

*Biomarkers* like fecal calprotectin and C-reactive protein to monitor disease activity, predict relapses, and guide treatment escalation or de-escalation.

*Nutritional management:* Identifying dietary triggers, risk of malnutrition, enteral and parenteral nutrition options, and post-surgical nutritional needs.

*Psychosocial considerations:* Addressing anxiety, depression, and coping mechanisms for chronic illness, particularly in adolescents transitioning to adult care.

*Reproductive health in IBD:* Educating patients on IBD management during pregnancy, the impact of medications on fertility, and safe treatment options for expectant mothers.

*Socio-cultural aspects of IBD:* IBD affects patients from diverse cultural backgrounds, each with unique dietary patterns, health beliefs, and traditional remedies that influence disease perception and management. Counsellors should be trained in culturally sensitive communication, recognizing religious, dietary, and societal factors that may impact treatment adherence and lifestyle choices.

###### Supervised training

Supervised training with experienced clinicians, IBD nurses, psychologists, and dietitians will help counsellors develop practical skills. Exposure to multidisciplinary teams, including colorectal surgeons, rheumatologists, dermatologists, and hepatologists, is likely to enhance their ability to provide comprehensive guidance to patients.

###### Assessment and certification

Candidates for IBD counsellor training should undergo both written and practical assessments to evaluate their competencies, with certification awarded upon successful completion. To support continuous professional development, ongoing education through conferences, workshops, and collaboration with specialists will be essential for counsellors to stay current with emerging research and therapies. Certification should be periodically renewed based on predefined criteria, ensuring the ongoing professional and personal growth of the counsellors.

### Confidentiality and ethics in IBD care

Counsellors must adhere to strict ethical standards, maintaining patient confidentiality before discussing sensitive health information. Ensuring a safe and supportive environment encourages open communication.

### Evaluating outcomes and improving practice

Regular patient feedback will help assess the effectiveness of counselling interventions, enabling adjustments to improve adherence, disease management, and quality of life. Metrics such as hospitalizations, medication adherence rates, and patient-reported outcomes will be used to refine counselling strategies and ensure continuous improvement in IBD care.

### Financing the IBD counsellor training program

Establishing a sustainable IBD counseling program requires a multi-stakeholder approach and a coordinated effort among governments, the private sector, and philanthropic entities. Governments should integrate IBD counselors into national health programs to ensure long-term sustainability. National and international IBD organizations can provide additional support, while philanthropic foundations and private healthcare providers can contribute through corporate social responsibility (CSR) initiatives. The pharmaceutical industry can play a key role by supporting the program through public-private partnerships (PPPs), enhancing treatment adherence and patient support.

## Call to action

To drive meaningful change in healthcare delivery, it is imperative to integrate IBD counselling into health systems. This will help bridge critical gaps in care, reduce the burden on overstrained healthcare infrastructure, and provide personalized treatment and continuous support for patients. Achieving this goal requires collaboration with global IBD organizations, governmental and non-governmental entities, and industry partners, with public-private partnerships playing a key role in ensuring sustainability and local adaptation.

A fundamental step in this endeavour is the establishment of structured certification programs for IBD counsellors. National and international IBD organizations must converge to develop standardized curricula that comprehensively cover IBD management while maintaining cultural competence and adaptability. Certification will enhance the credibility and quality of IBD counselling, fostering a skilled IBD workforce dedicated to improving patient outcomes. Existing training curricula, scope of practice frameworks, and accreditation models from Europe and North America provide valuable foundations that can be adapted for LMICs. Aligning these structures with global best practices will ensure that the role of IBD counsellor is tailored to the unique challenges of healthcare systems. Additionally, patient support groups play a vital role in making IBD counselling patient-centered and responsive to real-world needs. Given the cultural, social, dietary, and financial diversity within regions such as South Asia, Latin America, and sub-Saharan Africa, implementation of IBD counsellor programs must address the specific needs of different populations while maintaining a standardized curriculum framework.

The establishment of IBD counsellors will not merely be an advancement in healthcare, it will be a critical step towards global health equity, ensuring that patients worldwide have equitable access to comprehensive, multidisciplinary IBD care.

## Contributors

Arshdeep Singh, Arshia Bhardwaj, Riya Sharma, Vandana Midha, Ajit Sood.

1. Arshdeep Singh: Conception, Literature review, Writing-original draft, Writing-review and editing, Figures, Visualization, Approval of the final draft submitted.
2. Arshia Bhardwaj: Writing-original draft, Writing-review and editing, Figures, Visualization, Approval of the final draft submitted.
3. Riya Sharma: Writing-original draft, Writing-review and editing, Approval of the final draft submitted.
4. Vandana Midha: Resources, Supervision, Writing-review and editing, Approval of the final draft submitted.
5. Ajit Sood: Resources, Supervision, Writing-review and editing, Approval of the final draft submitted.

All authors read and approved the final version of the manuscript.

## Data sharing statement

No new data were created or analyzed in this study. Data sharing is not applicable to this article.

## Declaration of interests

Ajit Sood declares receiving speaker honorarium from Pfizer India and Takeda India. All other authors declare no conflicts of interest.
